# Supplementary material for: Structural and Social Determinants of Health Factors Associated with County-Level Variation in Non-Adherence to Antihypertensive Medication Treatment
Source: Int J Environ Res Public Health. 2020 Sep 14;17(18):6684. doi: 10.3390/ijerph17186684 (PMC7557537; doi:10.3390/ijerph17186684)
Supplement: Supplementary file 1 [file ijerph-17-06684-s001.pdf]

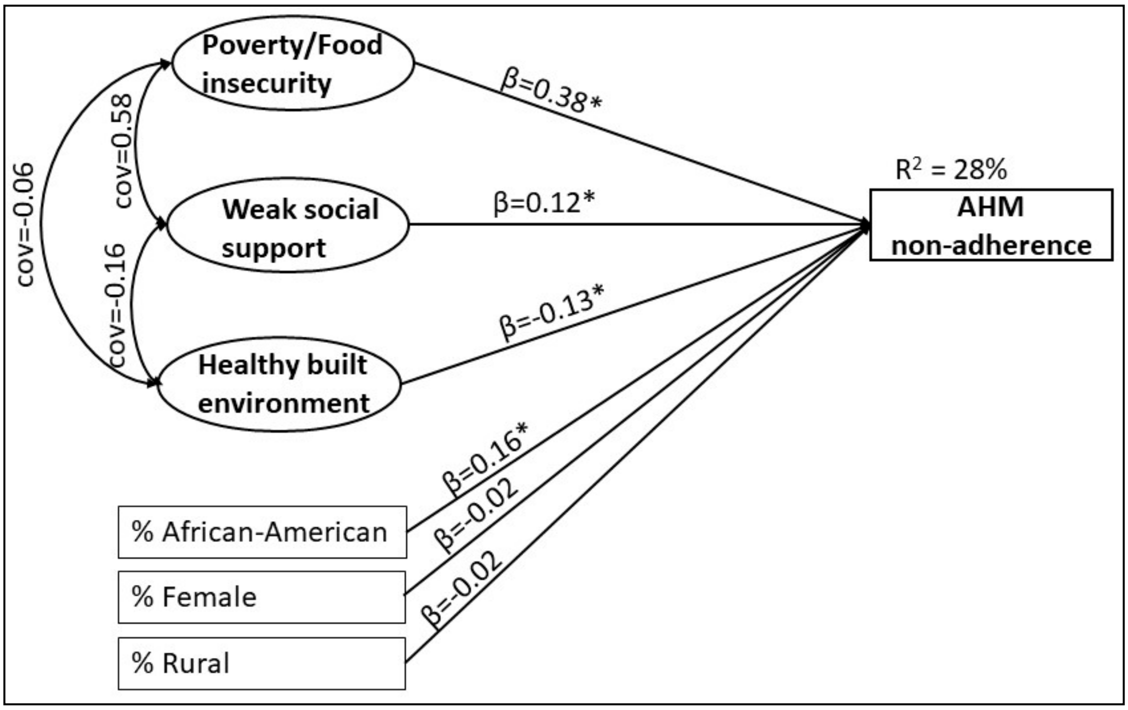

Figure S1: Relationships between social determinants of non-adherence and antihypertensive medication non-adherence adjusted for demographic factors. Indicators per construct. Poverty/food insecurity; Percent below poverty line (%), Uninsured (%), Food stamp/SNAP recipient (%), Food insecurity (%). Weak social support; Children in single-parent household (%), Families with female household head (%), Residential segregation, non-white/white (%). Healthy built environment; Population living within half a mile of a park (%).

Table S1. Summary statistics of model fit for hypothesized factor measurement and structural equation models.

	Chi-square	AIC	NFI	TLI	CFI
Hypothesized CFA measurement models					
Model 1	3860.40	3924.40	0.79	0.72	0.79
Model 2	2004.33	2060.33	0.81	0.72	0.81
Model 3	1969.84	2015.84	0.81	0.73	0.81
Hypothesized SEM (unadjusted)	2112.77	2166.77	0.81	0.73	0.81
Hypothesized SEM (adjusted)	10043.44	10125.44	0.59	0.52	0.59

Table S2. Factor loadings of indicator variables for social determinants of non-adherence constructs.

Constructs	β	Standard error	P-vale
**Poverty/food insecurity**			
*Percent below poverty line (%)	0.90	n/a	n/a
Uninsured (%)	0.44	0.02	<0.001
Food stamp/SNAP recipient (%)	0.90	0.02	<0.001
Food insecurity (%)	0.79	0.02	<0.001
**Weak social support**			
*Children in single-parent household (%)	0.86	n/a	n/a
Families with female household head (%)	0.80	0.02	<0.001
Residential segregation, non-white/white (%)	0.23	0.03	<0.001

Constructs	β	Standard error	P-vale
**Poverty/food insecurity**			
**Healthy built environment**			
*Population living within half a mile of a park (%)	0.79	n/a	n/a
Severe housing problems (%)	0.34	0.04	<0.001
Access to exercise opportunities (%)	0.64	0.05	<0.001

*The unstandardized loadings for each marker variable was constrained to a value of 1 to ensure model identification.
